# The role of GIPR in food intake control

**DOI:** 10.3389/fendo.2025.1532076

**Published:** 2025-03-17

**Authors:** Paula-Peace James-Okoro, Jo Edward Lewis, Fiona Mary Gribble, Frank Reimann

**Affiliations:** Institute of Metabolic-Science-Metabolic Research Laboratories and MRC-Metabolic Diseases Unit, University of Cambridge, Cambridge, United Kingdom

**Keywords:** Glucose-dependent insulinotropic polypeptide (GIP)/GIP-receptor (GIPR), Glucagon-like peptide-1 receptor (GLP1R), food intake, body weight, central nervous system, obesity, diabetes

## Abstract

Glucose-dependent insulinotropic polypeptide (GIP) is one of two incretin hormones playing key roles in the control of food intake, nutrient assimilation, insulin secretion and whole-body metabolism. Recent pharmacological advances and clinical trials show that unimolecular co-agonists that target the receptors for the incretins – GIP and glucagon-like peptide 1 (GLP-1) – offer more effective treatment strategies for obesity and type 2 diabetes mellitus (T2D) compared with GLP-1 receptor (GLP1R) agonists alone, suggesting previously underappreciated roles of GIP in regulating food intake and body weight. The mechanisms by which GIP regulates energy balance remain controversial as both agonism and antagonism of the GIP receptor (GIPR) produce weight loss and improve metabolic outcomes in preclinical models. Recent studies have shown that GIPR signalling in the central nervous system (CNS), especially in regions of the brain that regulate energy balance, is essential for its action on appetite regulation. This finding has sparked interest in understanding the mechanisms by which GIP engages brain circuits to reduce food intake and body weight. In this review, we present key knowledge around the actions of GIP on food intake regulation and the potential mechanisms by which GIPR and GIPR/GLP1R agonists may regulate energy balance.

## Introduction

Glucose-dependent insulinotropic polypeptide (previously known as gastric inhibitory peptide, GIP) is a gut-derived hormone produced and secreted from enteroendocrine K cells of the duodenum and upper jejunum upon meal ingestion (1). Along with glucagon-like peptide-1 (GLP-1), GIP plays a key role in regulating postprandial blood sugar levels through what is known as the *incretin effect* – where an estimated 50-70% of insulin release in response to a meal is mediated by GIP and GLP-1 (1). As the insulinotropic effect of GIP is diminished in patients with type 2 diabetes (T2D) (2), the therapeutic potential of the GIP axis has been relatively underexplored, whilst structurally optimised agonists of GLP-1 receptors (GLP1R) have been developed and introduced for the treatment of T2D and obesity. Focus is now turning to the therapeutic potential of targeting GIP receptors (GIPR), in light of the preclinical and clinical success of agents combining a GLP1R agonist with either a GIPR agonist or antagonist - strategies that deliver superior body weight reduction compared with GLP1R agonists alone (3–8).

Although research into the effects of GIP on appetite regulation is advancing, significant gaps remain in our understanding of the specific mechanisms underlying its anorectic action, particularly within the brain. Clarifying these central mechanisms is crucial, as the brain plays a vital role in regulating energy balance, and understanding how GIP affects central pathways to modulate food intake will be invaluable for improving GIP-based therapies for obesity and diabetes treatment. In this review, we present current knowledge on the role of GIPR signalling in the control of energy balance and examine emerging evidence regarding the potential mechanisms by which GIP affects food intake.

## The physiology of GIP

The mature form of GIP, GIP(1-42), is a 42-amino acid hormone derived from posttranslational processing of the 153-amino acid precursor, pre-pro-GIP, through prohormone convertase (PC) 1/3-dependent cleavage after Arg-65 in the proGIP sequence (9). GIP belongs to the secretin/glucagon family of structurally related neuroregulatory peptides, which also includes pituitary adenylate cyclase-activating peptide (PACAP) and growth hormone-releasing hormone (GHRH). Its amino acid sequence is highly conserved across species, showing over 90% sequence identity across human, murine, porcine and bovine species (1). GIP(1-42) is susceptible to rapid degradation and inactivation by dipeptidyl peptidase 4 (DPP4), the same enzyme that inactivates GLP-1, resulting in a short plasma half-life of 5-7 minutes (10).

GIP is primarily produced by enteroendocrine K cells in the upper small intestinal epithelium (11, 12). A shorter form, GIP(1-30), has been reported in α-cells of pancreatic islets by immunohistochemistry, reverse-transcription polymerase chain reaction (RT-PCR), and *in situ* hybridisation (ISH) (13). In contrast, we were unable to detect GIP-peptides by mass spectrometry in human or mouse islets, or *Gip*-mRNA in FACS-purified islet cell types from mice raised in our facility (14, 15).

## Expression of *Gipr* in peripheral tissues and the brain

The *Gipr* gene, while primarily known for its expression in pancreatic β-cells, is also found in other tissues, including adipose tissue, stomach, bone, adrenal cortex, heart, pituitary, endothelial cells, testis and several brain regions (1). Activation of GIPR thus exerts pleiotropic biological effects including neuroprotection (16), decreased bone resorption (17), and improved lipid metabolism and storage (18).

GIP was not historically considered to have any direct action on the brain, but intracerebroventricular (ICV) injection of high concentrations of GIP affected the secretion of anterior pituitary hormones including follicle-stimulating hormone (FSH) and growth hormone (GH), leading to the hypothesis that GIP could act on its receptors in hypothalamic regions near the third ventricle (19), and marking the first indication that GIP could have a regulatory role in the central nervous system (CNS). Subsequently, GIP receptors were identified in a rat cerebral cortex cDNA library and found to be similar to the receptors for glucagon and GLP-1, placing GIPR in the vasoactive intestinal polypeptide (VIP)/glucagon/secretin receptor superfamily (class B1) of seven transmembrane-domain G-protein coupled receptors (GPCRs) (20). GIP receptors primarily signal through Gαs/adenylyl cyclase activation, which increases intracellular cAMP levels; in pancreatic β-cells, this activates protein kinase A (PKA) and exchange protein activated by cAMP2 (EPAC2), resulting in a downstream increase in intracellular calcium levels and exocytosis of insulin (21–23).

Initial exploration of *Gipr* mRNA expression in the rat brain employing ISH and RT-qPCR revealed its wide distribution across many areas including the olfactory bulb, cerebral cortex, hippocampus, mammillary bodies, anterior and lateral septum, cortical amygdala, substantia nigra, thalamic nuclei, rostral raphe nuclei, choroid plexus, cuneate nucleus, cerebellum and brainstem (21, 24). In a study by Kaplan et al., autoradiographic localisation of saturable (25) GIP radioligand binding identified GIP binding sites in discrete areas of the rat brain, including the cortex, subiculum, anterior olfactory nucleus, inferior colliculus, lateral septal nucleus and the inferior olive (26). More recently, Adriaenssens et al. employed a *Gipr-*Cre knock-in mouse model, in which *Gipr*-expressing cells exhibit expression of a fluorescent EYFP reporter, to map *Gipr* expression in the mouse CNS (27). Immunostaining for EYFP highlighted *Gipr* expression in regions such as the medial preoptic area, subfornical organ, anterodorsal thalamic nucleus, magnocellular preoptic nucleus, suprachiasmatic nucleus and the interfascicular nucleus – alongside areas already identified in previous studies. Results from Cre-reporter lines must be interpreted with caution, as they can report cells expressing only very low levels of the receptor message, and might aberrantly report lineage tracing of cells that transiently expressed Cre-recombinase during development. Knock-in of Cre-recombinase into the native *Gipr*-locus, as in this model, should however reduce the risk of aberrant expression. Notably, *Gipr* mRNA expression was confirmed by qPCR in mouse hypothalamus and by ISH (RNAscope) specifically in the arcuate nucleus (ARH) and dorsomedial nucleus (DMH) of the hypothalamus of mice and several nuclei of the hypothalamus of humans (27). ISH (RNAscope) also confirmed expression of *Gipr* in the area postrema (AP) and nucleus tractus solitarius (NTS) of the brainstem in mice (28, 29) and Cynomolgus monkey (29). In the hindbrain, the signal in the AP was more pronounced compared to the NTS, suggesting that this area might be a particularly critical brain region for the regulation of energy balance and/or food intake regulation in response to GIPR-agonists.

Transcriptomic profiling using single-cell RNA sequencing of fluorescent cells isolated from *Gipr*-Cre mice further revealed heterogeneous expression of *Gipr* across neuronal and non-neuronal cell types in the hypothalamus. Based on the expression of cell-type-specific marker genes, *Gipr* was identified in mural cells, ependymocytes, pericytes, vascular and leptomeningeal cells (VLMCs), smooth muscle cells (SMCs), endothelial cells (ECs), oligodendrocytes (OLs), and neurons (27, 30). Analysis of the neuronal cluster showed *Gipr* expression in both glutamatergic and GABAergic neurons, along with co-expression of neurohormones involved in energy balance, including *Sst*, *Avp*, *Pthlh*, and fewer neurons expressing *Cartpt* and *Tac1 (* 27). More recently active *GIPR* expression in mouse and human hypothalamus was confirmed by single-nucleus (sn) RNA sequencing and spatial transcriptomics (31). *Gipr* neurons expressed receptors for key gut peptides known to regulate energy homeostasis such as ghrelin and cholecystokinin (CCK), and calcium imaging analysis demonstrated that stimulating these receptors excites *Gipr* cells, suggesting that *Gipr* neurons can respond to food-related signals from the periphery.

Histological and snRNAseq have characterised *Gipr* expression in the brainstem, revealing differential expression of *Gipr* within the NTS and AP (27, 32–34). *Gipr* expression is abundant in inhibitory GABAergic neurons within the AP, but less so in the NTS and the nodose ganglia of the vagus nerve (28, 35). Projections from inhibitory GABAergic neurons are mostly confined within the AP itself with minimal projections to the proximal NTS (32). Some studies additionally identified *Gipr* in a small population of glutamatergic neurons in the dorsal vagal complex (DVC) of the mouse brainstem (28), potentially reflecting the sparse *Gipr*-positive cells in the NTS that were also identifiable by RNAscope. Both GABAergic and glutamatergic *Gipr*-positive neurons were identified in snRNAseq analyses of the combined AP and NTS from rats and mice (36, 37). Non-neuronal cells, particularly OLs and a few astrocytes express *Gipr (* 33, 34, 37), revealing the multifaceted nature of *Gipr* distribution in the brainstem, as in hypothalamus, as outlined above.

## *Gip* expression in the CNS

The expression of *Gipr* and identification of GIP binding sites in the brain, particularly in regions protected by the blood-brain barrier (BBB), suggests that GIP potentially plays a physiological role in the CNS. This raises important questions about whether central *Gipr*-expressing populations respond to circulating GIP from the periphery or if there is a central population of GIP-producing cells. Early studies, which attempted to detect *Gip* mRNA in the rat brain by northern blot hybridisation, ISH and RT-PCR were unable to detect any *Gip* mRNA (21, 38, 39). Given this, GIP was suggested to enter the brain through circumventricular organs (CVO) such as the AP and act on other brain regions (21). Alternatively, it was suggested that another ligand might activate GIPR within the brain (21). Some studies, however, have reported GIP mRNA and protein in rat retina (40), hippocampus (41), olfactory bulb, cerebellar Purkinje cells, cerebral cortex, substantia nigra (24, 42), and striatum (43), with moderate expression in the amygdala, lateral septal nucleus, pretectal nuclei, thalamic reticular nucleus as well as in several nuclei in the thalamus, hypothalamus, and brainstem (24, 42). In these studies, GIP immunoreactivity and mRNA colocalised with the neuronal marker NeuN, but not the glial marker GFAP (24, 42), suggesting that *Gip*-expressing cells may be neuronal. However, efforts by our research group using a *Gip*-Cre-reporter model that readily labels *Gip*-expressing cells in the duodenum (44) have been unable to detect *Gip* expression in the brain (45, 46). Given these findings, there is ongoing debate about whether GIP is truly produced in the brain or if its central effects are mediated by circulating GIP arriving from the periphery, or if an alternative ligand engages central GIPRs. It is plausible that GIP produced by the gut might affect brain function, as peripherally injected GIP was detected in cerebrospinal fluid (CSF) collected from mice cisterna magna (47). The questions of whether GIP is produced centrally and the physiological role of GIPR located behind the BBB continue to be subjects of active investigation.

## Therapeutic GIPR agonism and GLP1R/GIPR dual agonism

Hormones released from the gut postprandially play key roles in regulating energy balance by modulating appetite and blood glucose levels (48), making them viable targets for the treatment of obesity and T2D. Among these, GLP-1 has gained significant attention for its ability to decrease body weight by inhibiting food intake, regulate glucose metabolism and improve renal and cardiovascular function (49, 50). This led to the development of GLP-1-based cardiometabolic medicines including liraglutide and semaglutide, which have shown clinical success in treating obesity (51–53). However, the use of GLP-1-based drugs comes with dose-dependent adverse effects, with up to 60% of patients reporting gastrointestinal (GI)-related issues. Furthermore, many patients struggle to reach their glycaemic and weight loss targets (54). This has spurred efforts to identify and develop agents that can enhance and complement GLP1R agonism.

An innovative approach in recent drug development is the design of unimolecular peptides that engage multiple receptors to improve therapeutic efficacy. Combining GLP1R agonists with activity against receptors for other hormones such as GIP, glucagon and amylin has shown promising results (3, 4, 55, 56). The approach stems from findings that co-treatment with GIPR and GLP1R agonists enhanced weight loss in diet-induced obese (DIO) mice (3, 4). Unimolecular GLP1R/GIPR dual agonists not only amplify the metabolic benefits of GLP-1 therapies but may also reduce common side effects, offering an effective strategy for managing obesity and T2D-related conditions (57, 58).

The first dual GLP1R/GIPR agonist, MAR709 (also known as NNC0090-2746) showed balanced *in vitro* activity at GIPR (EC_50_ = 3pM) and GLP1R (EC_50_ = 5pM) (3). In rodent models with genetic and diet-induced obesity, MAR709 produced greater weight loss and glycaemic improvements compared with pharmacokinetically matched GLP-1 treatments (3). In a phase 2b trial, the reductions in body weight and blood glucose in T2D patients treated with MAR709 at the single tested dose were similar to dose-titrated liraglutide (59). However, MAR709 was not further developed for commercial reasons (55).

A second GLP1R/GIPR coagonist, tirzepatide (previously called LY3298176, marketed as Mounjaro^®^ for T2D and Zepbound^®^ for obesity, Eli Lilly) is a 39-amino acid peptide acylated at the lysine 20 residue with a C20 fatty diacid. This acylation facilitates noncovalent binding to albumin, extending its half-life to approximately 160 hours in humans, compared with 19-25 hours for MAR709. Tirzepatide is designed as an imbalanced agonist, exhibiting greater affinity for human GIPR than for GLP1R. In signalling studies involving cell lines expressing *GIPR* or *GLP1R*, tirzepatide showed comparable potency to GIP in activating GIPR but was less potent than GLP-1 in activating GLP1R (4). In phase 3 clinical trials, tirzepatide demonstrated more effective reductions in body weight (approximately 20-22% weight loss with once-weekly 15 mg dosing) and glycated haemoglobin levels (up to 2.6%), along with overall greater improvements in lipid profiles compared with 1 mg semaglutide in T2D patients, although it should be noted that a higher dose of semaglutide (2.4 mg) is currently recommended for treating obesity (4, 51–53, 60, 61). It remains unclear whether the superior efficacy of tirzepatide in glucose and weight reduction, compared with the more balanced dual agonist MAR709, is due to its imbalanced pharmacology, its longer half-life or specific properties of tirzepatide such as biased GLP1R agonism (62). Collectively, these findings highlight the potential of GIPR agonism as a promising complementary target for achieving significant weight loss and blood glucose regulation, and there is growing interest in understanding how GIPR activation contributes to improved metabolic control and weight reduction (57).

As discussed in more detail below, a large body of preclinical research supports the idea that GIPR agonism plays a physiologically significant role in the mechanism of action of GLP1R/GIPR dual agonists. Mice fed a high-fat diet (HFD) showed improved body weight and glycaemic control when *Gip* was overexpressed (63). GIP analogues reduce appetite and lead to weight loss in DIO mice (64, 65), particularly when used in conjunction with GLP1R agonists. Importantly, cotreatment with agonists for GIPR and GLP1R reduced food intake and body weight to a greater extent than either agonist administered alone (3–6). These effects of GIP and GLP1R/GIPR dual agonists to reduce food intake suggest that GIP may act through central mechanisms and recruit neural networks that regulate energy balance and feeding behaviour.

## GIPR antagonism

Despite the clear success of agonising GIPR in the treatment of obesity, there is evidence that taking the opposite approach and antagonising GIPR activity could also be an effective treatment strategy. *Gipr* knockout mice fed a HFD show protection against obesity and insulin resistance even on a hyperphagic leptin-deficient background (66). Some studies have shown that high-fat diets increase intestinal K cell density in ob/ob mice, or reported elevated circulating levels of GIP in obese animals and humans (67–71). It remains to be shown whether elevated GIP levels seen in these reports drive further fat accumulation or reflect a failing counterregulatory response; deleting GIP in the context of leptin deficiency, however, had little effect on the development of obesity in ob/ob mice (72). Various GIP or GIPR pharmacologic inhibition strategies in rodents, including genetic ablation of GIP-secreting K cells, infusions of neutralising antibodies against GIP, monoclonal antagonistic antibodies against GIPR, and vaccination against GIP, have all been shown to protect against HFD-induced weight gain without substantially deteriorating glucose homeostasis (47, 73–81). However, it is worth highlighting that chronic antagonism or knockout of GLP1R produces similar protection against diet-induced obesity in mice (82–84), implying that both incretin hormones exhibit paradoxical agonist/antagonist effects.

One of the strongest arguments for developing therapeutic GIPR antagonists is that in humans, common (E354Q) (85) and rare (R190Q, E288G) (86) coding variants of *GIPR* that are associated with decreased receptor signalling (87–89) have been associated with lower body mass index (BMI). A recent study found that *GIPR* missense mutations resulting in a loss of both Gs-coupled cAMP accumulation and β-arrestin coupling are associated with a lower BMI, whereas selective loss of function of Gs-coupling was not “protective” in a similar manner, pointing towards an important role of β-arrestin signalling in GIPR-function (90, 91). Several groups are developing GIPR antagonists for the treatment of obesity, as discussed in more detail below. These promote weight loss in preclinical and/or clinical studies (7, 8, 47, 78, 92–94) and demonstrate superior weight loss when combined with GLP1R-agonism compared to GLP1R-agonism alone, but it remains unclear what mechanisms these agents engage.

**Figure 1:**
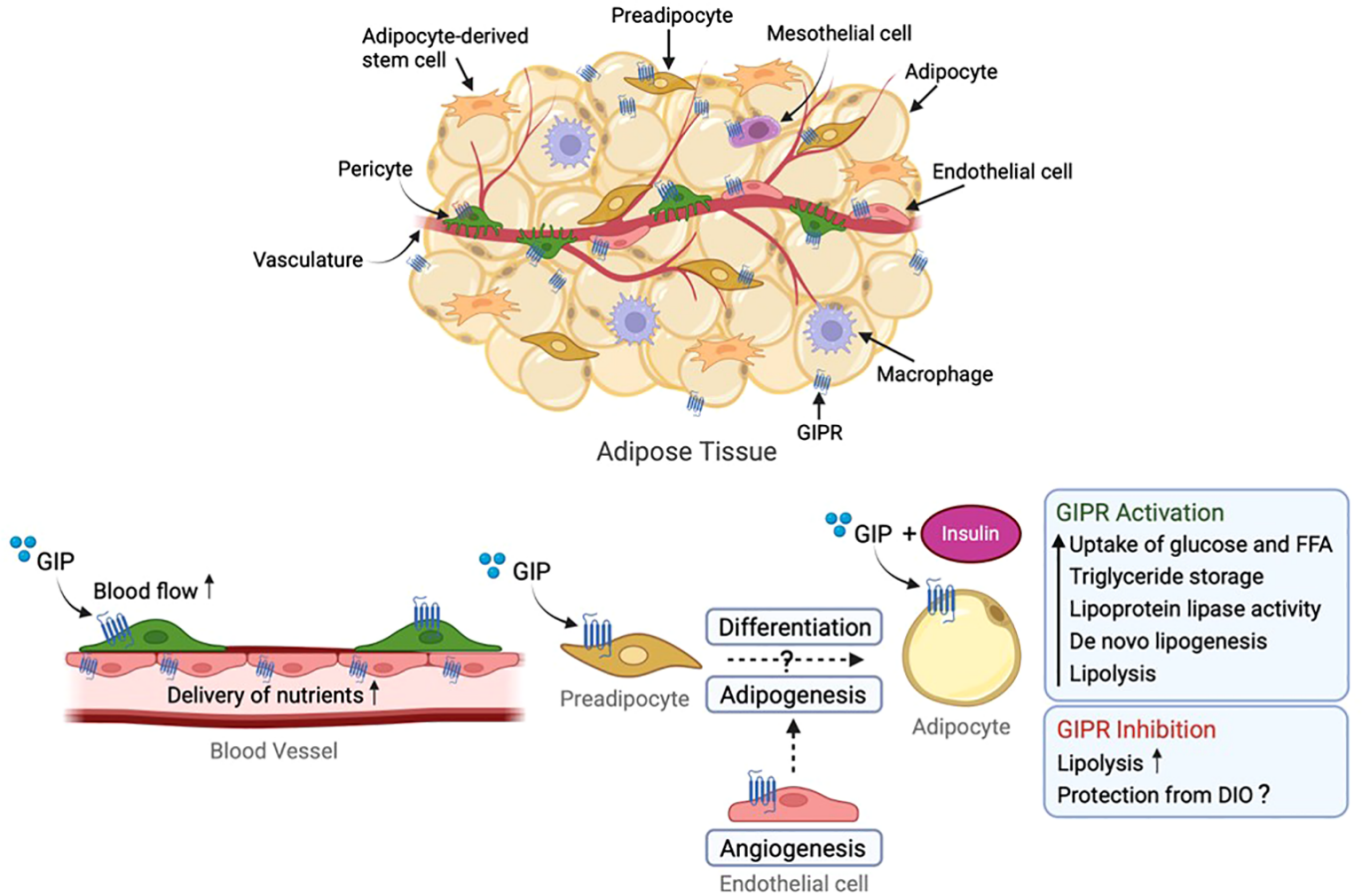
Role of GIP receptor in white adipose tissue (WAT). GIP receptor is expressed within adipose tissue in endothelial cells, macrophages, pericytes, mesothelial cells and adipocytes (the latter being discussed controversially). GIP stimulates blood flow and transport of nutrients to WAT, storage of triglycerides, lipoprotein lipase (LPL) activity, glucose and free fatty acid uptake, insulin sensitisation, *de novo* lipogenesis as well as lipolysis. Conversely, GIPR protects against HFD-induced weight gain presumably by inhibiting GIP-induced triglyceride storage and lipogenesis at the level of the adipose tissue. GIP, glucose-dependent insulinotropic polypeptide; GIPR, GIP receptor. Figure created with BioRender.com.

Some studies have suggested that increased energy expenditure is important for protection against DIO upon GIPR knockout or antagonism (73, 77, 81, 95, 96). Such studies consider effects on food intake to be secondary to weight loss, and emphasise the importance of altered glucose and lipid handling, possibly affected by altered postprandial insulin excursions (76). GIP mediates the uptake, storage and synthesis of fatty acids and triglycerides in adipocytes, especially under conditions of hyperinsulinemia (97–99) and it seems likely that GIPR-blocking agents and/or *Gipr* knock-out achieve some of their protection from diet-induced weight gain at the level of the adipose tissue. This lipogenic effect of GIP is thought to be mediated through mechanisms including increased secretion and activity of lipoprotein lipase (97, 100, 101), stimulation of insulin-dependent glucose uptake, increased adipocyte insulin receptor affinity, and conversion of glucose to lipids in the adipose tissue (98, 102, 103). Whilst the expression of *GIPR* in white adipocytes *in vivo* has been questioned (104), GIPR activation in combination with insulin does improve the lipid storage ability of adipose tissue by increasing responses to elevated insulin levels (105) whilst also improving lipid mobilisation when insulin levels are low (106) (**Figure 1**). It could be argued that inhibition of GIPR in adipose tissue interferes with its lipid buffering ability and that rodents, which as small animals are very sensitive to the insulating function of white adipose tissue, will under such circumstances increase their use of fatty acids to produce heat, e.g. increasing their energy expenditure. Consistent with this idea, *Gipr* knock-out mice have been reported to become obese on a HFD when kept at thermoneutrality (28°C) (107).

Other studies have pointed towards a direct effect of GIPR inhibition on food intake (7, 47). One study linked the anti-obesity effect of GIPR antagonism to leptin sensitivity in the hypothalamus, where genetically or pharmacologically blocking GIPR enhanced the anorexigenic properties of leptin in HFD-fed mice. Notably, *Gipr* knockout mice did not develop leptin resistance during HFD feeding (47). In the same study, it was also observed that central GIP could inhibit the appetite-suppressing effects of leptin. This GIP-induced leptin resistance occurred through activation of the small guanosine triphosphatase (GTPase) Ras-related protein 1 (Rap1), which inhibits the phosphorylation of signal transducer and activator of transcription 3 (STAT3) – a crucial mediator of leptin action (47, 108). This is, however, unlikely to be the only action of GIPR in the regulation of body weight, as whole-body *Gipr* knock-out also reduced body weight in the leptin-deficient ob/ob mouse model (66). It is also noteworthy that Rap1b activation downstream of the GIPR and Epac1 has recently been implicated in an anti-ageing action of GIP in the brain, reducing ferroptosis (109).

Emerging evidence suggests that the combination of antibody-based GIPR antagonism and GLP1R agonism leads to more significant weight loss in mice compared to the weight loss achieved through GLP1R agonism alone (7, 110). A bispecific molecule, maritide, in clinical trials comprises an antagonistic GIPR antibody coupled to two GLP-1 peptides. Phase 1 data on maritide, injected once monthly due to its long half-life, demonstrated effective body weight lowering in obese humans (8). The contribution of GIPR antagonism to the effectiveness of this molecule is not currently clear, as clinical dosing results in high circulating levels, raising the effective concentration of the GLP-1 component several orders of magnitude above the EC_50_ for GLP1R. As discussed later, GIP also acts to reduce nausea triggered by GLP-1, so antagonising GIPR could enhance the capability of the GLP-1 moiety to reduce food intake through the induction of nausea. An alternative approach of combining a GLP1R agonist with an antagonistic GIP peptide (AT-7687) is also under development, with some notable success in inducing weight loss in non-human primates (93).

Of note, whilst inhibition of food intake in response to peripheral treatment with a GIPR-blocking antibody was instantaneous in non-human primates, the effect took several days to develop in obese mice and might be secondary to weight loss (7). This group from Amgen also linked the effects of their GIPR-antagonistic antibody on body weight to adipose GIPR signalling and proposed that long-acting GIPR-agonism might act as functional antagonism due to receptor desensitisation/downregulation in adipocytes, but not other tissues such as pancreatic islets (92). This latter idea is, however, difficult to reconcile with reported effects of dual GLP1R/GIPR agonism which showed improved lipid flow into and out of adipose tissues, dependent on GIPR-agonism (106).

Effects of therapeutic GIPR antagonism should also be considered in the context of peripheral and central inflammation. Several studies suggest a pro-inflammatory role for GIP, contrasting with the well-documented anti-inflammatory action of GLP1R agonists (111, 112). Short term GIP infusion in human subjects increased IL-6 and monocyte chemoattractant protein-1 (MCP-1) in adipose tissue biopsies, likely reflecting enhanced macrophage-adipocyte crosstalk (113). Another study reported higher levels of inflammatory markers and differential expression of genes linked to leukocyte chemotaxis and tissue infiltration in obese adults with higher compared with lower fasting GIP levels, although participant numbers were relatively low and obesity parameters differed between the groups (114). In mouse models, central or peripheral administration of GIP promoted hypothalamic inflammation, with proinflammatory-related factors such as *Il-6* and *Socs3* elevated in the hypothalamus, which were reversed by an antagonistic GIPR antibody (115). By contrast, other studies reported anti-inflammatory effects of GIP, including a reduction in neuroinflammation surrounding amyloid plaques in a mouse model of Alzheimer’s disease after chronic treatment with [D-Ala_2_]GIP or a dual GLP1R/GIPR agonist. However, in these and other studies, it will be crucial to disentangle the extent to which inflammatory changes reflect direct effects of GIP on the inflammatory system rather than indirect effects arising from alterations in body weight and adiposity (116, 117).

## Gut-brain circuits in the regulation of food intake

The gut-brain axis is a communication network of neuronal, biochemical, and hormonal signals connecting the GI tract to the brain. Following meal ingestion, changes in circulating nutrient levels, stimulation of GI mechanoreceptors, and the release of gut hormones work together to regulate subsequent food intake (118). Peripherally-derived adiposity signals such as leptin and insulin, alongside short-term satiety signals such as GLP-1, CCK and peptide YY (PYY), interact with brain circuits to modulate feeding behaviour and maintain energy balance (119, 120). Information from the gut is relayed to the brain by vagal and splanchnic afferent pathways; whilst these express receptors for gut hormones, discussion of these is beyond the scope of this review, given that *Gipr* expression within the CNS seems to be crucial for GIP’s action on food intake (discussed in next section). In the brain, neurons in the hypothalamus and the brainstem are essential for the control of energy homeostasis and are well placed anatomically to mediate the effects of gut peptides on feeding [reviewed in (121)]. The hypothalamus, particularly the ARH, is strategically located adjacent to the third ventricle and the median eminence (ME) – a CVO with fenestrated capillaries, lacking a BBB and allowing access of peripheral nutritional signals to these appetite-controlling regions of the CNS (122). Similarly, the DVC is located near the fourth ventricle in the medulla oblongata. It includes the AP, NTS and the dorsal motor nucleus of the vagus (DMV), with the AP being another CVO accessible to peripheral signals due to its leaky BBB. AP neurons and vagal sensory neurons project directly to the NTS, enabling the NTS to integrate and transmit gut-derived information to the brain (123, 124). Gastrointestinal, circulatory and central cues integrate in the DVC, which projects to the hypothalamus to regulate response to the body’s energy status and influence feeding behaviours, and vice versa (125, 126). This gut-brain communication forms a crucial feedback loop for maintaining energy balance and adapting feeding behaviour based on physiological needs.

## Role of central GIPR signalling in appetite regulation

As apparent from the parallel development of drugs combining GLP1R agonism with either GIPR agonists or antagonists for the treatment of obesity, GIP appears to have several, sometimes opposing, actions on the brain. On the one hand, GIP signalling seems to favour weight gain, as evidenced by the protection of *Gipr* knockout mice from DIO, the lower BMI of humans with loss of function variants in GIPR, and a recently established ability of GIP to overcome nausea triggered by agents such as GLP1R agonists, discussed in more detail later. On the other hand, GIPR agonists have been demonstrated to have direct inhibitory effects on food intake in mice – a finding that is less evident in humans – and dual GLP1R/GIPR agonists in clinical practice trigger more weight loss than GLP1R agonism alone. These combined findings suggest that GIPR agonists have more than one action on the brain, potentially acting on different neuronal populations with different effects depending on the feeding status. Before embarking on a discussion of the different GIPR-containing neuronal circuitries identified in the literature, it should be acknowledged that a unifying consensus incorporating the multiple observations surrounding GIP biology has not yet been reached.

Owing to the rapid inactivation of endogenous GLP-1 and GIP by DPP4, it is currently debated whether endogenous gut-derived incretins reach their brain receptors at concentrations high enough to exert physiological actions. We recently demonstrated inhibition of food intake in response to activating intestinal K-cells that could be blocked by either systemic or central administration of a GIPR-blocking antibody (46). However, other studies failed to observe significant effects on food intake in mice when activating K-cells (25, 127). Degradation-resistant incretin receptor agonists undoubtedly have potential to act directly on the brain, likely through the CVOs, which are accessible to peripherally-administered fluorescent GIPR and GLP1R ligands (28, 128–130).

The expression of *Gipr* in feeding centres of the hypothalamus and brainstem suggests that GIP and GIP-based agonists could engage neuronal and/or non-neuronal circuitry in these regions to regulate food intake and body weight (27, 29, 30, 33, 64, 131). Emphasising the importance of brain GIPR signalling, deletion of *Gipr* from the CNS replicates the protection from DIO observed in global germline *Gipr* knockout models (64). Selective removal of *Gipr* from GABAergic cells using *Vgat-*Cre-dependent recombination further pointed to the importance of GABAergic neurons for both the protection against DIO and the reduction in food intake in response to systemically applied GIPR-agonists, especially when co-administered with a GLP1R-agonist (129, 132).

Both the brainstem and hypothalamus have been considered the primary central detectors of GIP and GIPR agonists in the peripheral circulation, since both areas contain neurons expressing *Gipr* within or adjacent to regions with a leaky BBB. Overlap of c-Fos staining and *Gipr*-Cre reporter-positive cells after administration of peripheral GIPR-agonist was more pronounced in the brainstem (particularly the AP) than the hypothalamus (46). Application of GIP also triggered cAMP elevation and increased neuronal activity as measured by electrophysiological recordings in hindbrain slices (133). A role for AP *Gipr*-positive neurons, the majority of which are GABAergic (32), does not, therefore, seem to be in any doubt, but whether this cell population underlies all the observed effects of GIPR agonists is less clear. A study by Adriaenssens et al., which characterised the distinct roles of *Gipr*-expressing neurons in the hypothalamus and brainstem, demonstrated that these neuronal populations engage different anorexigenic pathways depending on their neuroanatomical location (28). Whilst activation of *Gipr*-neurons in either region reduced dark-phase food intake, chemogenetic stimulation of DVC but not hypothalamic *Gipr* neurons induced conditioned taste aversion (CTA) (28). As evidence from other studies shows that AP *Gipr* neurons are anti-aversive, as discussed below, the researchers suggested that the CTA induced by DVC *Gipr* chemogenetic stimulation is mediated by direct activation of *Gipr*-neurons deeper in the NTS rather than the AP, and that the NTS is not a primary target of peripherally-administered GIPR agonists.

**Figure 2:**
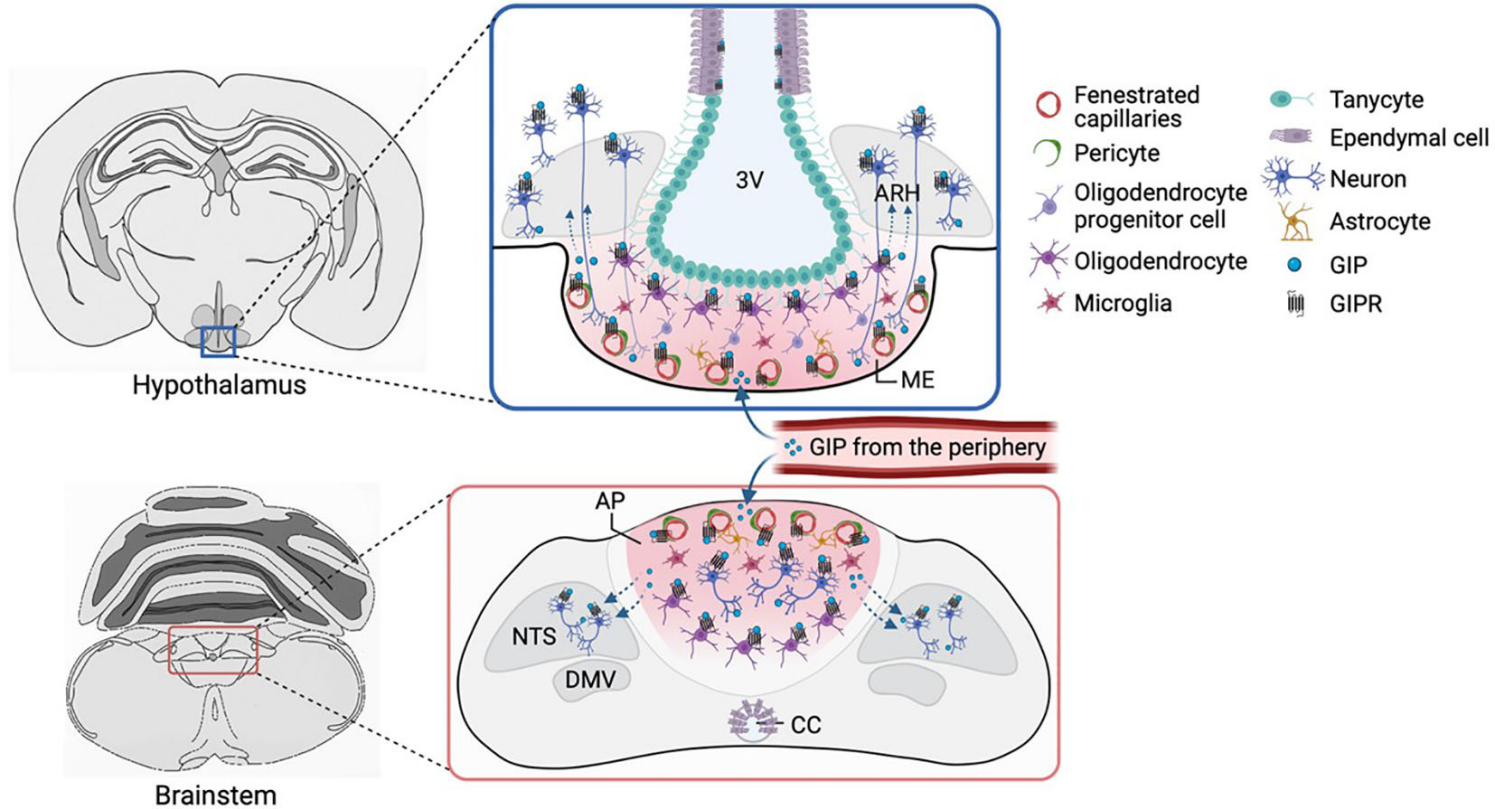
GIPR expression in the hypothalamus and brainstem. *Gipr* is expressed in oligodendrocytes, pericytes and other vascular and glial cells that control and regulate the transfer of circulating factors from CVOs (such as the ME and AP) to the brain parenchyma. Whilst GABAergic neurons in the AP have cell bodies, dendrites and axons outside the BBB, such neurons are scarce in the ME, although axons or dendrites from cells in adjacent areas (such as the ARH) might reach the ME, potentially allowing these neurons access to plasma-borne hormones. Alternatively, *Gipr* expression on non-neuronal cells might affect access to neuronal receptors behind the BBB. 3V, third ventricle; ARH, arcuate nucleus; ME, median eminence; CC, central canal; BBB, blood-brain barrier; CVO, circumventricular organs. Figure created with BioRender.com.

The weight loss and food intake suppressing effects of long-acting GIPR agonists and the GIP component of GLP1R/GIPR co-agonists have been attributed to inhibitory GABAergic neurons, likely reflecting *Gipr*-neurons in the AP (129) (**Figure 2**). In DIO WT mice, acyl-GIP and MAR709 decreased food intake, body weight and fat mass, concomitant with an increase in neuronal activation in the AP. Deletion of *Gipr* in *Vgat*-expressing GABAergic neurons blocked the effect of acyl GIP on AP neuronal activity, food intake, and body weight reduction, and abrogated the superior effectiveness of MAR709, suggesting that GIPR signalling via inhibitory GABAergic neurons is necessary for GIP’s anorectic effects (129). Further studies are necessary to clarify whether acyl-GIP and GLP1R/GIPR co-agonists act exclusively on brainstem GABAergic GIPR neurons or whether they also act on GABAergic GIPR neurons in other brain regions to reduce body weight and food intake.

Of note, mice lacking *Gipr* in *Vgat*-expressing neurons were hypersensitive to GLP1R-agonist, losing more weight than wild-type animals in response to the same GLP1R-agonist dose (132). Similar GLP1R-agonist hypersensitivity was observed in a previous study when GIPRs were selectively removed by stereotactic injection of rAAV-Cre into the hypothalamus of *Gipr* ^fxfx^ mice (28). However, this manipulation failed to prevent the additional food intake reduction that results from adding a GIPR-agonist on top of treatment with a GLP1R-agonist, suggesting that the hypothalamus is not the major site of GIPR-agonist action for food intake reduction, despite the reduction in food intake triggered by chemogenetic activation of hypothalamic *Gipr*-expressing neurons in the *Gipr*-Cre model (27).

In addition to the strong brainstem c-Fos signal triggered by administration of GIP analogues, this intervention also labels key hypothalamic regions implicated in feeding regulation, including the ARH, PVH, DMH, ventromedial (VMH), and lateral hypothalamus (LH) (29, 33, 46, 64, 129, 134). This suggests that hypothalamic engagement is involved in the overall neuronal circuitry important for and may underscore the action of GIP on food intake reduction. However, the overlap of c-Fos in response to peripheral GIPR-agonist administration and *Gipr*-Cre dependent reporter expression was limited in the hypothalamus (46),consistent with the idea that hypothalamic engagement occurs downstream of hindbrain activation by GIP. The relevance of the detected *Gipr* mRNA and the *Gipr*-positive cells reported by the *Gipr*-Cre model in the hypothalamus (**Figure 2**) therefore remains debated. Whilst it could be argued that these cells represent aberrant *Gipr*-Cre activity, the detection of *Gipr* mRNA in Hypomap argues against this (31). Alternatively, hypothalamic cells express *Gipr* at levels too low to elicit functional responses, or are not accessible to GIPR-agonists in the circulation; direct measurements of functional responses to GIP in *Gipr*-expressing neurons in brain slice preparations, which could potentially distinguish between these options, are currently lacking. However, it should be noted that cAMP or Ca^2+^ responses could be absent from a *Gipr*-labelled cell soma if the GIPR-containing compartment has been lost in the brain slice preparation or if the receptors are located in small, restricted compartments such as presynaptic terminals where they could play an important role in sensitising to other stimuli. Further studies will be crucial to disentangle this important area.

Regardless of exactly where GIP is primarily detected by the brain, the hypothalamus seems important in its downstream effects on food intake. Hunger-promoting AgRP neurons in the hypothalamus are necessary and sufficient to drive food intake and are key in maintaining energy balance (135–139), and although *Gipr* is not expressed in hypothalamic AgRP neurons themselves, GIPR and GLP1R agonism acutely inhibit AgRP neuronal activity in fasted mice, reducing their responses to food (140). In this context, it may be relevant that the ARH receives direct projections from the NTS to regulate feeding (141–143), and GABAergic projections from the DVC inhibit AgRP/NPY neurons in the ARH to decrease food intake and body weight (144). Studies by Kaneko and colleagues investigating the mechanism of action of intracranially-administered GIPR-antagonism alternatively implicated hypothalamic POMC neurons in underlying GIP-induced leptin resistance (47). However, another study involving systemic administration of the GIPR-agonist, GIPFA-085, suggested that increased plasma leptin levels recruit hypothalamic POMC-neurons in the ARH to underlie the reduced food intake (145). GIP’s interaction with leptin thus remains contentious, and additional research is needed to clarify the role of GIP in leptin signalling as well as to provide a more comprehensive understanding of the role of *GIPR*-expressing neurons in the hypothalamus in general.

## GIP reduces nausea and emesis via the brainstem

The gut hormones, GLP-1 and PYY, reduce food intake by targeting overlapping neuronal circuits in the hypothalamus and brainstem that reduce appetite and trigger nausea and emesis (146–148). Despite their profound metabolic success, GLP-1-based medications cause nausea and vomiting in many patients (146–148). These GI side effects are considered a barrier to maximising the weight-loss profile of these treatments, suggesting a need to develop and test the efficacy of novel agents with improved tolerability. Interestingly, GIPR agonists have been shown to inhibit emetic responses caused by cisplatin, GLP-1 or PYY in dogs, ferrets and musk shrews, as well as nausea-associated behaviour in mice and rats (29, 33, 36, 134, 149). These findings have led to the concept that GIP acts as an anti-aversive hormone in response to GI-derived nauseating signals (134) and that GIPR agonism may improve the weight loss effectiveness of GLP1R agonists partly by reducing GLP1R agonist-induced nausea, allowing higher dosing of the GLP1R agonist.

**Figure 3:**
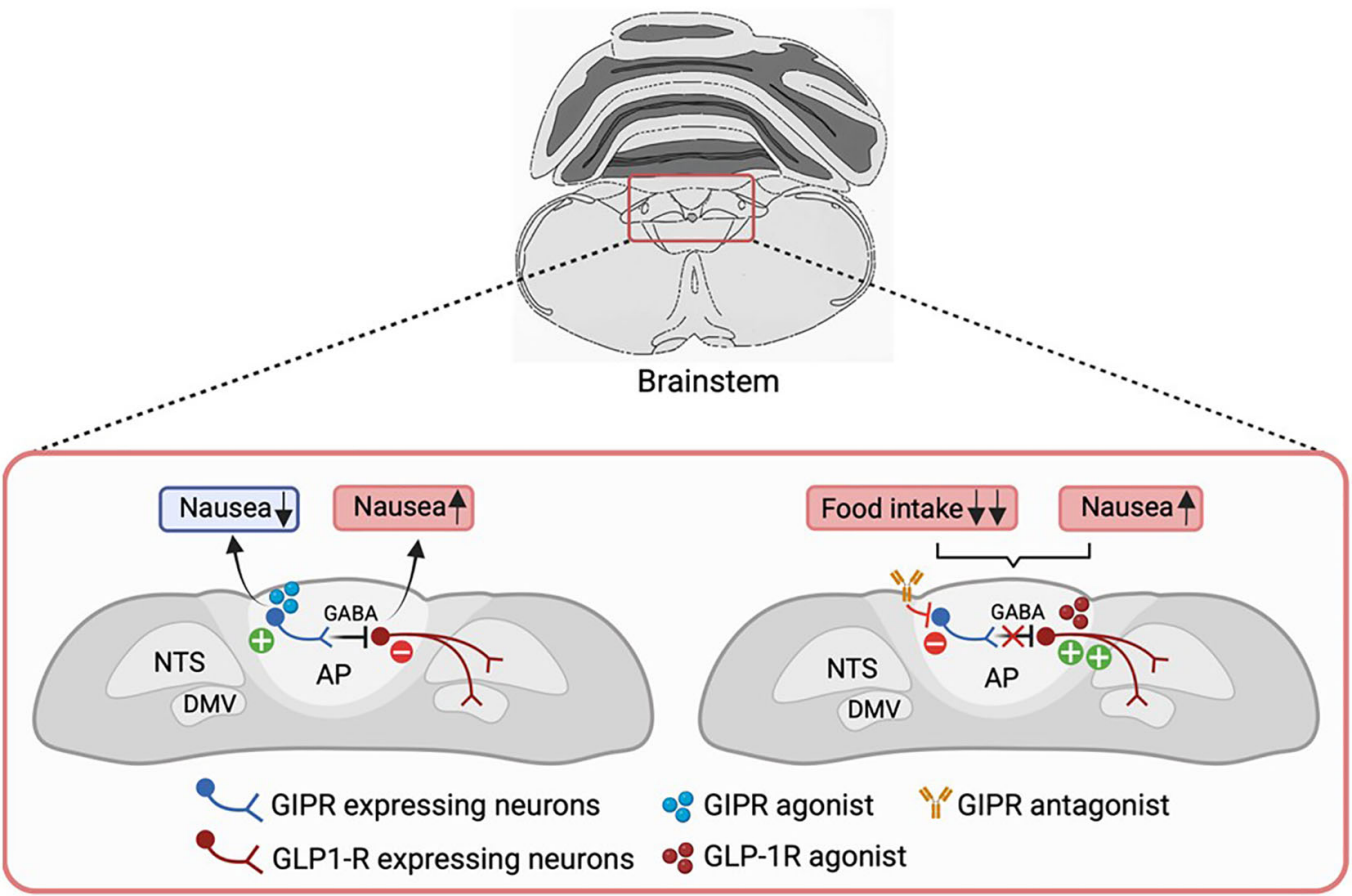
GIPR agonism in modulating GLP1R-induced nausea. Activation of GIPR might alleviate GLP-1-induced malaise by directly acting on the AP/NTS circuitry. *Gipr* is expressed in a subset of GABAergic inhibitory neurons in this region, through which GIP may act to suppress the activity of nausea-promoting GLP1R neurons, thereby reducing nausea and improving the therapeutic index of GLP-1-based medications. It remains to be seen if the same GABAergic GIPR-expressing neurons also underlie food intake inhibition by GIPR-agonists. Conversely, GIPR antagonism suppresses the activity of these inhibitory neurons, leading to enhanced activation of GLP1R-expressing neurons. This results in a greater reduction of food intake, albeit likely with increased nausea as a side effect of GLP1R activation. GABA, gamma-aminobutyric acid; AP, area postrema; NTS, nucleus of the solitary tract; DMV, dorsal motor nucleus of the vagus; GIPR, GIP receptor; GLP1R, GLP-1 receptor. Figure created with BioRender.com.

The AP contains inhibitory neurons that counteract nausea and vomiting responses triggered by certain visceral stimuli (150–152), and as discussed above, *Gipr* is expressed in a subset of GABAergic inhibitory neurons in this region. GIP has been demonstrated to activate these inhibitory neurons, and thereby suppress the activity of nausea-promoting excitatory neurons, including those which express the growth/differentiation factor 15 (GDF15) receptor (*Gfral*), *Glp1r (* 29) and the calcium-sensing receptor (*Casr*) (32, 133) (**Figure 3**). Diphtheria toxin-mediated ablation of AP *Gipr* neurons eliminated the anti-nausea effects of GIP (133). However, it is unknown whether these anti-aversive effects of GIP reflect its physiological as well as its pharmacological actions, and future studies will be required to determine whether intestinal GIP plays a physiological role in inhibiting avoidance responses to nauseating gastrointestinal signals.

## GIP may influence BBB permeability and brain access to peripheral factors

For GIP- and GLP-1-based therapies to exert their full anorectic effects, they must access their receptors located in CNS regions that modulate energy balance. In the majority of brain regions, the BBB carefully controls the entry of molecules from the bloodstream into the brain [reviewed in (153)]. Fluorescent GIPR and GLP1R agonists dosed peripherally can directly access brain areas that lie outside the BBB. This includes the choroid plexus and CVOs, which display a compromised BBB. CVOs with particular interest for the regulation of food intake include the ME of the hypothalamus and the AP of the brainstem (28, 128). Fluorescently labelled agonists were also detected, albeit at low levels, in brain regions supposed to be protected by the BBB, including the posterior ARH, NTS and DMV, which are adjacent to the ME and AP, respectively, and have been implicated in the regulation of food intake (28, 128). Radiolabelled GIP was additionally found to cross the BBB in a time-dependent saturable manner, which was inhibited by competition with native unlabelled GIP (26), suggesting involvement of a receptor-mediated mechanism, and GIP has been detected in the CSF of mice (47) and humans (154), which is interesting in the context of reported evidence of a neuroprotective effect of GIP in animal models for Alzheimer’s and Parkinson’s disease (16, 155).

While the majority of metabolic sensing neurons located in the ARH are separated from the ME by diffusion barriers, these neuronal populations may extend dendrites or axons to the ME or contact the ME through axonal terminals and directly access peripherally secreted hormones (122, 156–158). Tanycytes are thought to participate in the formation of CVO-parenchymal barriers (159, 160) and peripheral liraglutide has been shown to enter the hypothalamus via transcytosis across tanycytes (161). However, there is currently no similar evidence for GIP crossing into the brain through this mechanism; *Gipr* expression is especially high in ependymal cells surrounding the third ventricle (31), and oligodendrocytes but future work will need to address if these are involved in regulating access of hormones in the plasma to neurons behind the BBB. Enhancing the permeability and diffusion of these molecules to neuronal populations deeper in the brain that regulate energy balance may be a potential strategy to maximise the weight-loss effect of GLP1R/GIPR agonists (153, 162). Further research is needed to clarify the central mechanisms and routes through which these drugs reach their target sites in the brain, and the importance of brain penetration for induction of weight-loss.

Notably, in the hypothalamus and brainstem, *Gipr* is expressed by vascular and glial cells which affect perfusion and permeability, including pericytes, smooth muscle cells, vascular and leptomeningeal cells, endothelial cells and oligodendrocytes (27, 30, 37, 163). Oligodendrocytes are highly responsive to the nutritional state, as fasting triggers rapid proliferation and differentiation of OLs (74). Transcriptomic and FISH analyses of the murine and human hypothalamus have demonstrated that the *Gipr* is enriched in oligodendrocytes, particularly in the ME (27, 30, 31, 163–165). Supporting a functional role of GIPR signalling in the regulation of the oligodendrocyte lineage, a recent study demonstrated that oligodendrocyte-specific *Gipr* deletion in adult mice reduced oligodendrocyte survival and oligodendrogenesis, whilst treatment with GIPR agonists enhanced oligodendrocyte plasticity in the ME (165). GIPR signalling in oligodendrocytes increased access of GLP1R agonists to ME axons, and boosted the anorectic and weight-loss effects of GLP1R agonism (165), suggesting a novel mechanism by which GIPR agonism may enhance the weight loss profile of GLP1R agonists in incretin-based therapies.

## Conclusion and future perspectives

The success of dual incretin receptor agonists as obesity pharmacotherapies has reignited interest in the previously overlooked role of GIPR signalling in energy balance. In addition to its insulinotropic effects, it is increasingly clear that GIP plays a role in modulating food intake and body weight, likely through central pathways that are not yet fully elucidated. GIP analogues act on regions of the brain that regulate energy balance, nausea and feeding behaviour to reduce food intake. Anti-emetic effects of GIP may enhance the therapeutic efficacy of GLP1R/GIPR dual agonists by mitigating the nauseating effects of GLP1R agonism – although this is yet to be proven in humans. Given the success of GLP1R/GIPR dual agonists in clinical trials, further understanding how GIPR signalling in the CNS affects energy balance is vital to fully harness the therapeutic benefits of GIP to combat obesity and T2D.
